# Protein Arginine Methyltransferase 5 (PRMT5) Mutations in Cancer Cells

**DOI:** 10.3390/ijms24076042

**Published:** 2023-03-23

**Authors:** Shayaan Rasheed, Renee A. Bouley, Ryan J. Yoder, Ruben C. Petreaca

**Affiliations:** 1 James Comprehensive Cancer Center, The Ohio State University Columbus, Columbus, OH 43210, USA; 2 Biology Program, The Ohio State University, Columbus, OH 43210, USA; 3 Department of Chemistry and Biochemistry, The Ohio State University, Marion, OH 43302, USA; 4 Department of Molecular Genetics, The Ohio State University, Marion, OH 43302, USA

**Keywords:** post-translational modification, arginine methylation, mutation, cancer

## Abstract

Arginine methylation is a form of posttranslational modification that regulates many cellular functions such as development, DNA damage repair, inflammatory response, splicing, and signal transduction, among others. Protein arginine methyltransferase 5 (PRMT5) is one of nine identified methyltransferases, and it can methylate both histone and non-histone targets. It has pleiotropic functions, including recruitment of repair machinery to a chromosomal DNA double strand break (DSB) and coordinating the interplay between repair and checkpoint activation. Thus, PRMT5 has been actively studied as a cancer treatment target, and small molecule inhibitors of its enzymatic activity have already been developed. In this report, we analyzed all reported PRMT5 mutations appearing in cancer cells using data from the Catalogue of Somatic Mutations in Cancers (COSMIC). Our goal is to classify mutations as either drivers or passengers to understand which ones are likely to promote cellular transformation. Using gold standard artificial intelligence algorithms, we uncovered several key driver mutations in the active site of the enzyme (D306H, L315P, and N318K). In silico protein modeling shows that these mutations may affect the affinity of PRMT5 for S-adenosylmethionine (SAM), which is required as a methyl donor. Electrostatic analysis of the enzyme active site shows that one of these mutations creates a tunnel in the vicinity of the SAM binding site, which may allow interfering molecules to enter the enzyme active site and decrease its activity. We also identified several non-coding mutations that appear to affect PRMT5 splicing. Our analyses provide insights into the role of PRMT5 mutations in cancer cells. Additionally, since PRMT5 single molecule inhibitors have already been developed, this work may uncover future directions in how mutations can affect targeted inhibition.

## 1. Introduction

Post-translational protein modifications are essential for the efficient function of proteins and enzymes. Most modifications concerning decorating proteins with sugars occur in the endoplasmic reticulum and Golgi apparatus [1,2]. Other forms of post-translational modification that occur outside of the ER and Golgi modulate protein or enzyme function [3].

Arginine methylation is one form of modification with pleiotropic effects including DNA damage repair, splicing, cell cycle regulation, signal transduction, etc. [4,5,6,7,8]. Nine different protein methyltransferases have been identified [5] and are generally divided into three groups: (1) monomethylators that add one methyl group onto arginine; (2) symmetric dimethylators that methylate both arginine side-chain nitrogen groups, also known as Type II; and (3) asymmetric dimethylators that methylate the same arginine side-chain nitrogen group, also known as Type I [5]. PRMT5 is a symmetric (Type II) di-methylating enzyme [9]. 

Protein methyltransferase mutations have been identified in various cancers, suggesting that dysregulating this type of post-translational modification is important for cellular transformation and immortalization [10,11,12]. PRMT5 is one of the methyltransferases initially identified in yeast with pleiotropic functions in humans, including in development and cancer [13,14]. PRMT5 methylates both histone targets and non-histone targets [15]. Related to DNA damage repair, PRMT5 methylates histones H2A, H4, and H3 in human cells. One key non-histone target of PRMT5 in humans is the Ruvbl1 subunit of the KAT5 complex, which is methylated at R205 [16]. Ruvbl1 methylation is required for recruitment of the KAT5 complex to the breaks via interaction with histone H3K9me3. KAT5 acetylates several residues of histone H4, including H4K16 [17,18,19,20,21], which promote an open chromatin structure and facilitate DNA double-strand break repair [22,23,24]. 

PRMT5 also regulates other pathways in the cell, and mutations and even mislocalization of this protein have been detected in cancers [15,25]. A PubMed search with the prompt “PRMT5 cancer” retrieves over 500 articles highlighting the importance of this gene in cancer. Consequently, PRMT5 small molecule inhibitors are already in clinical trials [6,15]. High expression of PRMT5 correlating with poor prognosis has been detected in many cancers, suggesting that PRMT5 has oncogenic function [26,27,28]. 

In this report, we queried the Catalogue of Somatic Mutations in Cancers (COSMIC) [29,30] and characterized all PRMT5 mutations. We employed two validated algorithms (CHASM and VEST4) [31,32,33,34] to classify mutations in terms of driver or pathogenicity. We then used various in silico protein modeling techniques to predict the effects of key mutations on PRMT5 structure and function. 

## 2. Results and Discussion

### 2.1. PRMT5 Mutation Spectrum in Cancer Cells 

**Figure 1:**
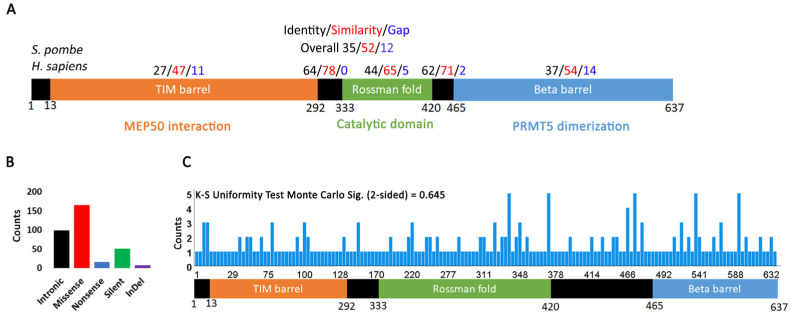
PRMT5 mutation distribution in human cancers. (**A**) Structure of PRMT5 protein showing conservation between *S. pombe* and *H. sapiens*. Residue conservation between the two species was determined using the NCBI Protein-BLAST tool. (**B**) Distribution of different types of PRMT5 mutations in human cancers. (**C**) Frequency of PRMT5 mutations occurring in the various protein regions. A one-sample Kolmogorov-Smirnov (K-S) test for uniformity was performed using SPSS. The Monte Carlo two-sided significance value is 0.645, indicating that mutations distribute uniformly over the PRMT5 region.

The longest and most common isoform of PRMT5 is 637 amino acids. It was identified as a JAK2 interacting protein in a two-hybrid screen and determined by sequence comparison to be the homologue of *S. pombe skb1^+^* and *S. cerevisiae HSL7* [35]. The crystal structure and further sequence analysis revealed the presence of three major domains [8,11,36,37,38] (Figure 1A). A TIM barrel domain at the N-terminus interacts with MEP50, a WD40 protein that facilitates PRMT5 interaction with its targets [39]. The catalytic domain is characterized by a Rossman fold required for interaction with S-adenosylmethionine (SAM) and transfer of methyl groups from SAM onto target residues [36]. The C-terminal domain is a beta barrel involved in PRMT5 dimerization [40]. A comparison of the human PRMT5 sequence with the *S. pombe skb1^+^* homologue showed that there is high conservation in the catalytic and dimerization domains (Figure 1A and Figure S1). The N-terminal TIM barrel region is less conserved, but this is not unexpected since MEP50 does not have a yeast homologue.

The Catalogue of Somatic Mutations in Cancer (COSMIC) [41] reports 338 total PRMT5 mutations (239 coding, 99 non-coding) in various cancers, with most being missense (Figure 1B, Supplementary Table S1. Interestingly, of all PRMT1-9 genes, only PRMT5 is characterized by a higher percentage of missense mutations, indicating that alterations in the PRMT5 coding sequence are more common in cancers (Supplementary Figure S2). This may suggest that PRMT5 plays a greater role in cellular transformation and cancer progression than the other PRMT genes. There is no statistically significant skew (e.g., hotspot) in the distribution of coding mutations (K-S test for uniformity *p* value = 0.645) (Figure 1C). PRMT5 mutations have been reported in most cancers, but the highest incidence is in skin cancer and the second highest in colorectal cancer (Supplementary Figure S3, Supplementary Table S1). One group reported that PRMT5 is upregulated in melanoma, which correlates with an increase in histone de-methylation and cellular proliferation [42]. Indeed, over 75% of skin cancers reported on COSMIC are malignant melanoma (Supplementary Table S1). PRMT5 mutations also play a major role in cellular transformation and the pathogenicity of colorectal cancers [43]. The other PRMT genes are also highly mutated in skin and colorectal cancers (Supplementary Figures S2 and S3). A quick PubMed search for literature on protein arginine methyl transferases and colorectal or skin cancers reveals that most research has been devoted to understanding the function of PRMT5, with sporadic publications on the functions of the other arginine methyltransferases. Our analysis suggests that mutations in all PRMT genes contribute to the development of these cancers.

**Figure 2:**
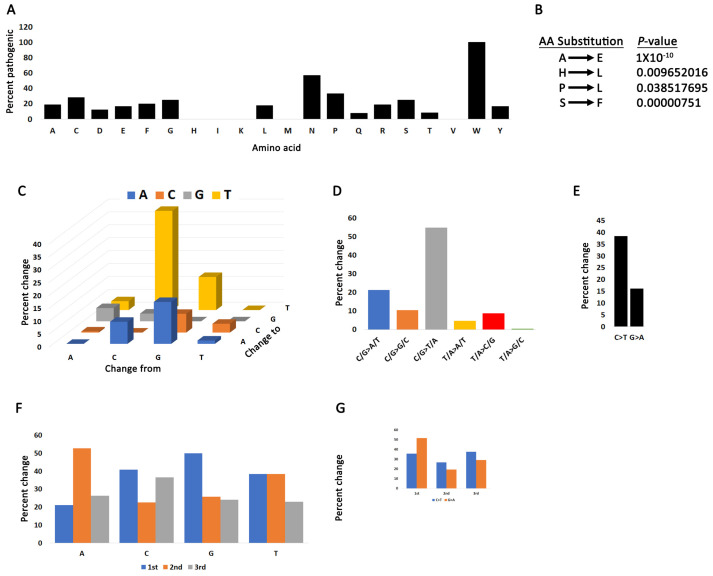
Mutation bias in the PRMT5 coding region. (**A**) The percent of substitutions for each amino acid that produce pathogenic mutations. (**B**) PRMT5 amino acid changes that have a statistically significant deviation from rates of change in human cancers. Chi-square test between PRMT5 and values reported by Anoosha et al. [45]. (**C**) Base pair substitutions in PRMT5. (**D**) Mutation spectrum of PRMT5 base pair substitutions in cancer cells. C/G > T/A transitions dominate the PRMT5 mutation landscape. (**E**) C > T transitions are the most prominent form of mutation. (**F**) The percent substitution of the four nucleotides occurring in the 1st, 2nd, or 3rd codon position. (**G**) C > T and G > A substitutions are more likely to occur in the first codon position.

Because not all mutations impact the function of the gene equally, we employed two algorithms to classify PRMT5 mutations as driver, pathogenic, or both. CHASMPlus provides statistical significance for predicted driver mutations [32,33] and VEST4 predicts the probability of mutations being pathogenic [34]. The CHASM or VEST4 artificial intelligence programs calculate the probability of any mutation being driver or pathogenic and generate a *p*-value. Any mutation with a *p*-value below 0.05 has the potential to be driver or pathogenic. Additionally, in a recent report, it was shown that CHASM is the best artificial intelligence program for categorizing COSMIC mutations [44]. We identified 64 mutations that are either drivers or pathogenic (Supplementary Table S1). The highest percent of pathogenic substitutions occur in tryptophan (W) residues (Figure 2A and Figure S4A,B). Anoosha et al. have previously determined the genome-wide frequency of amino acid substitutions in human cancers [45]. We compared the PRMT5 amino acid substitution frequency with genome-wide cancer frequency and identified four PRMT5 substitutions that deviate (significant Chi-square *p*-value) (Figure 2B). All substitutions with a significant value (Figure 2B) occur at a lower frequency than predicted by Anoosha et al. At the nucleotide level, substitutions are driven primarily by C > T and G > A transitions (Figure 2C–E and Figure S5, Supplementary Table S1). C > T transitions may be indicative of “clocklike signatures” which represent mutations accumulating with age [46]. C/T > G/A transitions also characterize the other PRMT genes (Supplementary Figure S4B,C). There is also a higher frequency of nucleotide substitutions from C and T in the first codon position, indicating that there is a greater chance of amino acid substitutions from mutations in these bases (Figure 2F–G and Supplementary Table S1).

**Figure 3:**
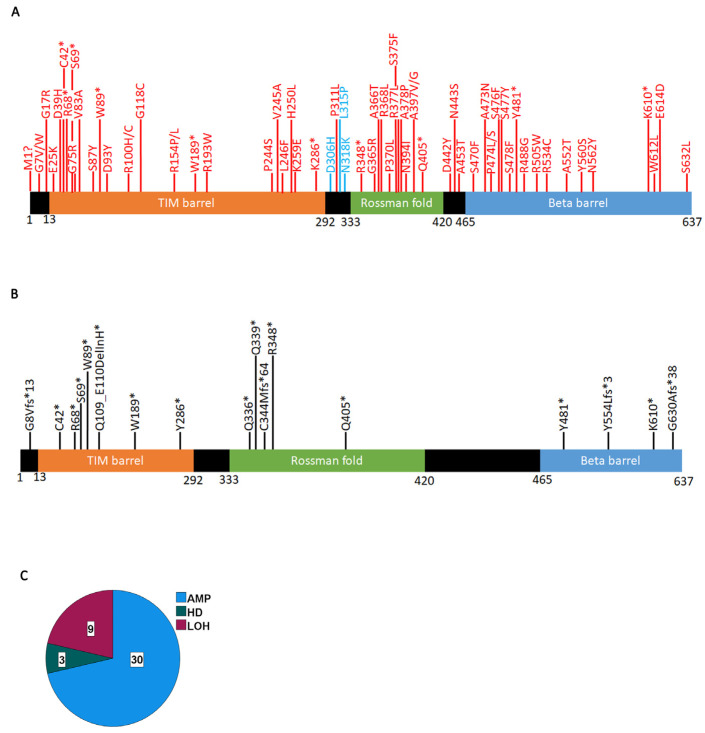
Significant PRMT5 mutations occurring in cancer cells. (**A**) Mutations with a significant VEST4 value (red) or significant VEST4 and CHASM values (blue) (Supplementary Table S1). (**B**) Truncating PRMT5 mutations (*) that are likely to affect the function of the gene. (**C**) PRMT5 locus structural variations. The data extracted from COSMIC using the CONNAN function.

We next mapped all PRMT5 mutations with significant VEST4 and CHASM values (Figure 3A, Supplementary Table S1). Pathogenic mutations (significant VEST4 value) distribute throughout all regions of the protein. Remarkably, three predicted driver mutations (D306H, L315P, and N318K) occur in the linker between the MEP50 interacting region (TIM barrel) and the catalytic site (Rossman fold). These mutations appear to decrease the affinity of PRMT5 for SAM (see the next section). The computational algorithms do not always make accurate predictions for frameshift and certain truncating mutations. However, these mutations are predicted to significantly affect the structure and function of the protein, so we also mapped these mutations (Figure 3B). We find that all identified truncating mutations occur in the three functional domains, with the highest concentration in the N-terminus TIM barrel, which truncates a major part of the protein. This suggests that most truncating mutations have a profound effect on PRMT5 function. COSMIC provides information on zygocity for a subset of mutations, and we find that all predicted driver or pathogenic mutations for which data are available are heterozygous, suggesting that complete inactivation of PRMT5 in cancer cells is rare. In fact, a pan-cancer analysis of copy number alterations shows that most often the PRMT5 region is amplified (Figure 3C). This agrees with findings that PRMT5 behaves like an oncogene [47,48,49] and consequently is overexpressed in most cancers [14]. We found three cases with PRMT5 homozygous deletions: a 76-year-old female with lung squamous cell carcinoma and two patients with kidney papillary renal cell carcinoma, an 82-year-old male and a 60-year-old female.

### 2.2. PRMT5 Mutation Effects on Enzyme Structure and Function

We next used in silico protein structure analysis to investigate the impact of the three identified driver and pathogenic mutations (D306H, L315P, and N318K) on PRMT5 protein structure and function. Each mutation was mapped on the available crystal structure of wild-type PRMT5 (PDB ID: 4GQB) [36] through Coot 08.9.3 [50].

**Figure 4:**
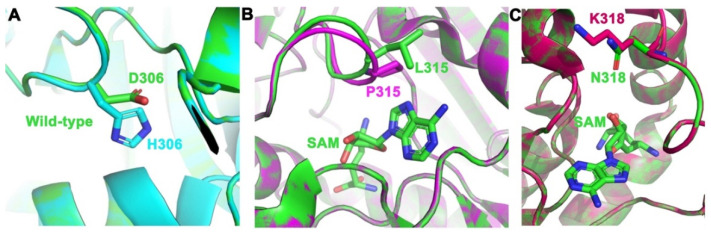
PRMT5 mutations were placed in their respective binding pockets. (**A**) Mutation of D306H (green to cyan) in the substrate-binding pocket. (**B**) Mutation of L315P (green to magenta) in a SAM binding pocket with SAM bound (green sticks). (**C**) Mutation of N318K (green to red) in a SAM binding pocket with SAM bound (green sticks). Figure was made by aligning the wild-type structure (green) of PRMT5 (PDB ID: 4GQB) [36] to the mutant structures using PyMOL.

PRMT5 possesses a substrate-binding site and an S-adenosylmethionine (SAM) binding site, both of which are crucial to the activity of this enzyme (Figure 4). The SAM cofactor acts as a methyl donor in the methyltransferase reaction, and as a result, changes to this site can have drastic effects on the activity of the enzyme. The three mutations were mapped on the enzyme to determine their locations relative to both the SAM site and substrate site of PRMT5. Of our three predicted driver mutations, L315P was found to be directly in the SAM site of PRMT5 (Figure 4B). The interaction of L315 within the SAM site has been previously described [51]. Additionally, the mutation N318K was found to be at the SAM site, and the D306H mutation was mapped next to the substrate site (Figure 3A,C). This indicates that the three mutated residues could affect PRMT5’s affinity for SAM or substrate, and thus, the ability of the protein to function normally. 

**Figure 5:**
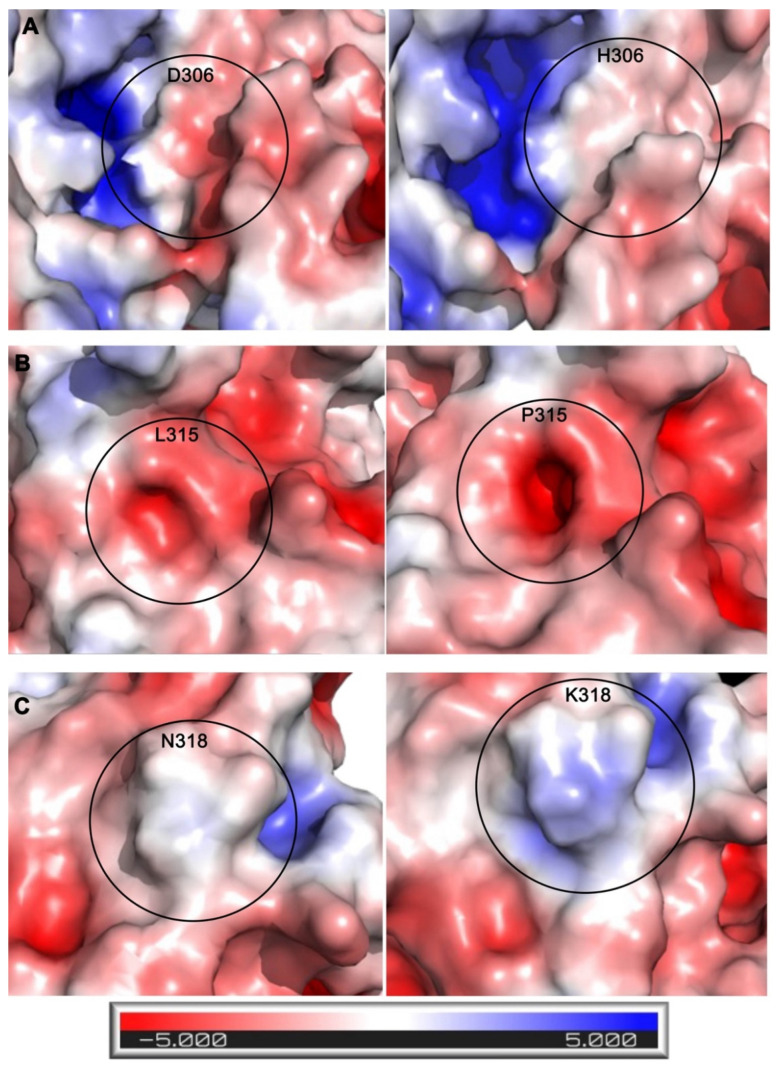
Electrostatic comparisons between wild-type (left panels) and mutated PRMT5 (right panels) residues. Comparisons between (**A**) D306H wild type and mutant, (**B**) L315P wild type and mutant, and (**C**) N318K wild type and mutant. Electrostatic surface potentials were calculated and displayed in PyMOL using the APBS plug-in. This is shown using a surface representation where red is a negative charge, blue is a positive charge, and white is neutral. The electrostatic scale is shown at the bottom, ranging from −5 to +5 kT/e.

To characterize how these mutations might affect protein function, the electrostatic surface potential of the wild-type protein was compared to each mutant (Figure 5). When analyzing the electrostatic models, increasing blue color represents increasing basicity of a region, increasing red color represents increasing acidity of a region, and the color white represents a neutral/hydrophobic region. The changes caused by the mutations can be qualitatively observed in Figure 5. In the mutant structure of D306H, there is an evident decrease in acidity next to the substrate site, changing the region to a neutral/hydrophobic area (Figure 5A). For the L315P mutation, there were no significant changes in charge or hydrophobicity between wild-type and mutant. However, the introduction of the proline mutation did create a change in the structure, which formed a tunnel towards the PRMT5 SAM site (Figure 5B). This tunnel was measured to be approximately 6 Å wide and extended approximately 8 Å deep into the SAM site. This resulting gap, then, could be sizable enough for small molecules to enter the SAM site, thus potentially allowing interfering molecules to enter the enzyme. When comparing wild type to the N318K mutation, there is a shift from a nearly completely neutral region by the SAM site to a substantially basic region at the point of mutation (Figure 5C). 

**Figure 6:**
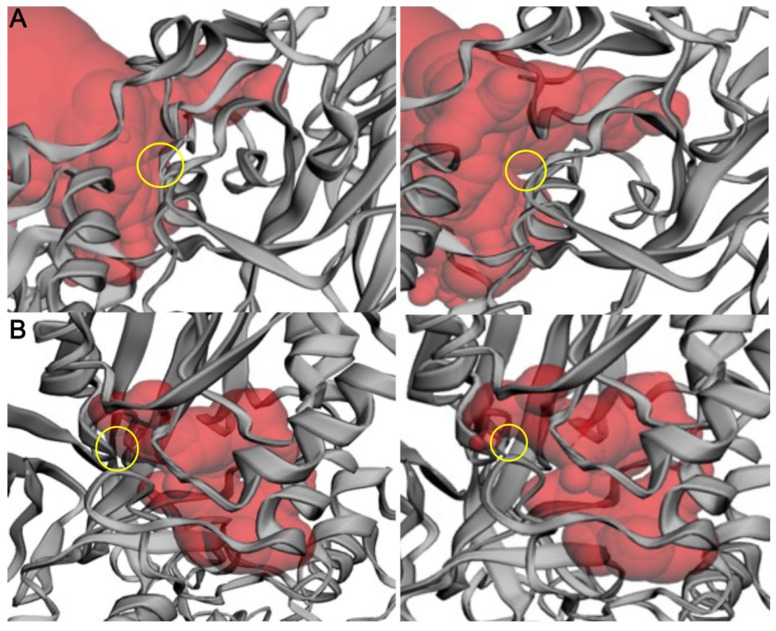
Change in the CASTp calculated binding pocket of wild-type PRMT5 versus mutants. (**A**) Wild type (left) and D306H mutant (right). (**B**) Wild type (left) and L315P mutant (right). Comparison of the available binding pocket (red) between the wild-type (left) and the mutant (right). The site of mutation + available binding pocket shift is indicated by the yellow ring. Protein structure is illustrated by the gray ribbon. Structure and pocket visualization were directly pulled from the CASTp server.

To further characterize the effect of these mutations, we calculated the effects of each mutation on binding pocket area (Å^2^) and volume (Å^3^) (Figure 6). We performed pocket analysis of the substrate-binding and SAM-binding pockets using CASTp, using the wild-type enzyme (PDB: 4GQB) (Table 1) [36]. The wild-type enzyme had a calculated area and volume of 1190.662 Å^2^ and 1458.760 Å^3^, respectively, in the substrate binding pocket and an area of 250.827 Å^2^ and a volume of 106.076 Å^3^ in the SAM co-factor binding pocket. Of the three mutations studied, two showed a change in area and volume of the respective pocket. The D306H mutation provided an increase in the available area and volume in the substrate binding site (Figure 6A). D306H incurred a 15.53% increase in area and a 7.94% increase in volume. There was no change for this mutation in the area and volume of the SAM binding site, confirming the location of this mutation in the substrate binding pocket. On the contrary, L315P demonstrated a reduction in available area and volume at the SAM binding site. L315P incurred a 10.47% reduction in area and an 8.28% reduction in volume (Figure 6B). There was no change in the substrate biding pocket area or volume for this mutation, further indicating its placement in the SAM site. N318K demonstrated little to no changes in accessible area and volume within either binding pocket (Supplementary Figure S6). The N318 residue is located at the edge of the SAM binding pocket (Figure 4C), which is consistent with these results. 

These mutations were also analyzed to determine if they would cause a destabilizing effect on protein structure using CUPSAT, an open-source program that utilizes amino acid-atom potentials and torsion angle distribution to predict changes to overall stability (Table 2) [52]. The overall torsion angle combination is shown as either favorable or unfavorable for the mutation. The predicted ΔΔG is used to determine whether the mutation would be destabilizing or stabilizing to the protein structure compared to the wild-type. This value represents the difference in the free energy of unfolding between wild-type and mutant cells, where a negative value indicates a destabilizing effect and a positive value represents a stabilizing effect. For all the possible single point mutations in PRMT5 calculated by CUPSAT, the ΔΔG values ranged from 29.06 to + 18.96 kcal/mol as the most destabilizing and stabilizing, respectively. Therefore, none of the three mutations analyzed here are predicted to significantly impact the structure and stability of PRMT5.

Given these initial modeling results, it certainly appears that these identified mutations may affect the structure of the PRMT5 binding pockets, which could in turn affect their ability to carry out their native function. Specifically, the L315P mutation within the SAM binding pocket showed the largest reduction in the volume of the binding pocket, in addition to creating other topographical differences. Future modeling studies (molecular docking, molecular dynamics, etc.) could further elucidate the potential effect of these mutations on the crucial function of PRMT5.

### 2.3. PRMT5 Non-Coding Mutations 

Non-coding mutations have been traditionally considered not to contribute to disease because they occur in “junk” DNA. However, more recent evidence suggests that these mutations do indeed have the potential to be pathogenic because they may affect splice sites or gene regulation [53]. Non-coding mutations fall into two types: (1) 5′ and 3′ untranslated regions (UTRs), which may affect transcription or mRNA stability, and (2) intronic mutations which may affect splicing but may also affect translation and mRNA stability [54]. COSMIC reports several PRMT5 non-coding mutations, both in the 5′ and 3′ UTRs as well as within introns (Supplementary Table S1). Several algorithms have been developed for analyzing non-coding mutations. A recent report has evaluated these algorithms on various data, including COSMIC data [55]. This evaluation found that the DANN algorithm [56] performs best for ClinVar data, while the FATHMM-MKL [57] algorithm was better for COSMIC data. We used both algorithms, and indeed, FATHMM-MKL provides a more stringent dataset of potentially pathogenic mutations than the DANN algorithm (Supplementary Table S1). Using the more stringent FATHMM-MKL analysis, we identified six splice site variants that are likely to be pathogenic (Table 3). FATHMM-MKL produces a score between zero and one with values closer to one predicted to be pathogenic with the highest confidence. We note that the caveat to these algorithms is that predictions are not yet possible for all loci as of the writing of this report because training data is still being developed [57].

All of identified predicted pathogenic mutations affect splicing (Table 3). The c.450 + 2T > C was previously reported in gastric cancers [58], while the 1c.1762-1G > A in endometrial cancers [59]. To our knowledge, the other mutations have not been described in the literature. Our analysis here suggests that PRMT5 splicing mutations are likely to destabilize the function of the enzyme and potentially drive cancer.

## 3. Discussion

Arginine methylation serves important physiological functions in the cell. PRMT5 has emerged as a major player in cellular transformation and cancer evolution. The pan-cancer analysis of PRMT5 mutations described in this report showed that not all mutations have an equal impact on cancer. The PRMT5 mutations were categorized as pathogenic or driver mutations using CHASM and VEST. Three driver mutations were analyzed and predicted to have a profound effect on the PRMT5 enzymatic activity. Given that PRMT5 small molecule inhibitors have already been developed, our in-silico analysis reveals potentially important directions in investigating the role of these mutations in targeted inhibition. 

We are aware of the limitations of this study in that it is only a predictive method for potentially pathogenic or driver mutations. The natural next step is to experimentally analyze some of these mutations. Nevertheless, the data presented here is a critical first step in that direction and is informative for further studies.

## 4. Materials and Methods

### 4.1. PRMT5 Mutational Analysis from COSMIC

Excel files (.csv) were downloaded from the COSMIC database (https://cancer.sanger.ac.uk/cosmic, accessed on 15 August 2022) version 96 on 4 August 2022. The OPEN-CRAVAT (Cancer-Related Analysis of Variants Toolkit) interface was used to classify mutations in terms of pathogenicity or driver [60,61]. The CHASMPlus tool [31,33] was used for driver classification and the VEST4 tool [34] for pathogenicity classification. The analysis presented in Figure 1A was done using the NCBI Protein-BLAST tool. Mutation analysis and statistics were performed in Excel or SPSS under an Ohio State University license. All figures were made in Photoshop.

### 4.2. In Silico Mutation Modeling

For in silico protein structure/function analysis, PRMT5 mutations were mapped on the wild-type crystal structure (PDBID: 4GQB) [36] through Coot 08.9.3 [50]. Local-geometry refinement was performed to minimize steric clashes, and the optimal rotamer was selected. The mutants were aligned to the wild-type structure in PyMOL version 2.3.4 to generate Figure 4. Electrostatic surface potential calculations were performed using the PDB2PQR server using the PARSE forcefield. The PB equation can be used to determine the electrostatic potential within and around a biomolecule by solving the partial differential equation [62]. The electrostatic surface potential was visualized using the Adaptive Poisson-Boltzmann Solver (APBS) software [62], which was developed to solve the equations of continuum electrostatics for large biomolecular assemblages [62]. The changes that occurred electrostatically were observed in PyMOL [63], using the APBS electrostatic plug-in to generate Figure 5. 

### 4.3. Enzyme Pocket Area and Volume Analysis

To calculate the volume of the binding pockets, each mutant enzyme structure was run through the Computed Atlas of Surface Topography of proteins (CASTp) [64] analytical program to visualize and calculate changes. CASTp uses the alpha shape method developed in computational geometry to identify topographic features, measure area and volume, and compute imprint. The pockets were visualized in PyMOL to generate Figure 6. 

### 4.4. Calculation of Mutant Stability

To calculate whether mutations were destabilizing to the protein structure, the webserver CUPSAT was used to calculate the ΔΔG, which represents the difference in the change in free energy for the unfolding of the wild-type compared to the change in free energy for the unfolding of the mutant [52,65,66]. The torsion angles for the mutation are also compared to the wild type to determine if this would be a favorable or unfavorable change. 

## Figures and Tables

**Table 1:** CASTp pocket analysis of PRMT5 mutations.

Enzyme Form	Substrate Binding Pocket Area (Å^2^)	Substrate Binding Pocket Volume (Å^3^)	SAM Binding Pocket Area (Å^2^)	SAM Binding Pocket Volume (Å^3^)
Wild-type	1190.66	1458.76	250.83	106.08
D306H	1375.55	1574.62	250.83	106.08
L315P	1190.66	1458.76	224.56	97.30
N318K	1190.66	1458.76	250.33	105.89

**Table 2:** CUPSAT analysis of PRMT5 mutant stability.

Mutation	Overall Stability	Torsion	Predicted ΔΔG (kcal/mol)
D306H	Destabilizing	Favorable	−0.96
L315P	Stabilizing	Unfavorable	+0.08
N318K	Stabilizing	Unfavorable	+0.37

**Table 3:** Likely pathogenic non-coding PRMT5 mutations.

Mutation Coordinates	FATHMM-MKL Score	Mutation Type	Tissue
c.564-1G > T	0.94646	splice_site_variant	Large intestine, lung
c.1199 + 1G > A	0.97494	splice_site_variant	Endometrium
c.450 + 2T > C	0.98287	splice_site_variant	Stomach
c.1762-1G > A	0.98355	splice_site_variant	Endometrium
c.614-1G > T	0.98663	splice_site_variant	Prostate, endometrium
c.1697-2A > G	0.99432	splice_site_variant	Large intestine, endometrium

## Data Availability

Data were obtained from the COSMIC database, which is freely available for non-commercial users. The analyzed data presented in this study are available in Supplementary Table S1.

## Supplementary Materials

The following supporting information can be downloaded at: https://www.mdpi.com/article/10.3390/ijms24076042/s1.
