# Supplementary material for: Protein Arginine Methyltransferase 5 (PRMT5) Mutations in Cancer Cells
Source: Int J Mol Sci. 2023 Mar 23;24(7):6042. doi: 10.3390/ijms24076042 (PMC10094674; doi:10.3390/ijms24076042)

**Supplementary Figure S1. Alignment of *S. pombe* and *H. sapiens* PRMT5 sequences.** Protein BLAST (https://blast.ncbi.nlm.nih.gov) was used to generate the alignments. Red arrows represent positions of mutated residues with significant CHASM and VEST4 probability values.

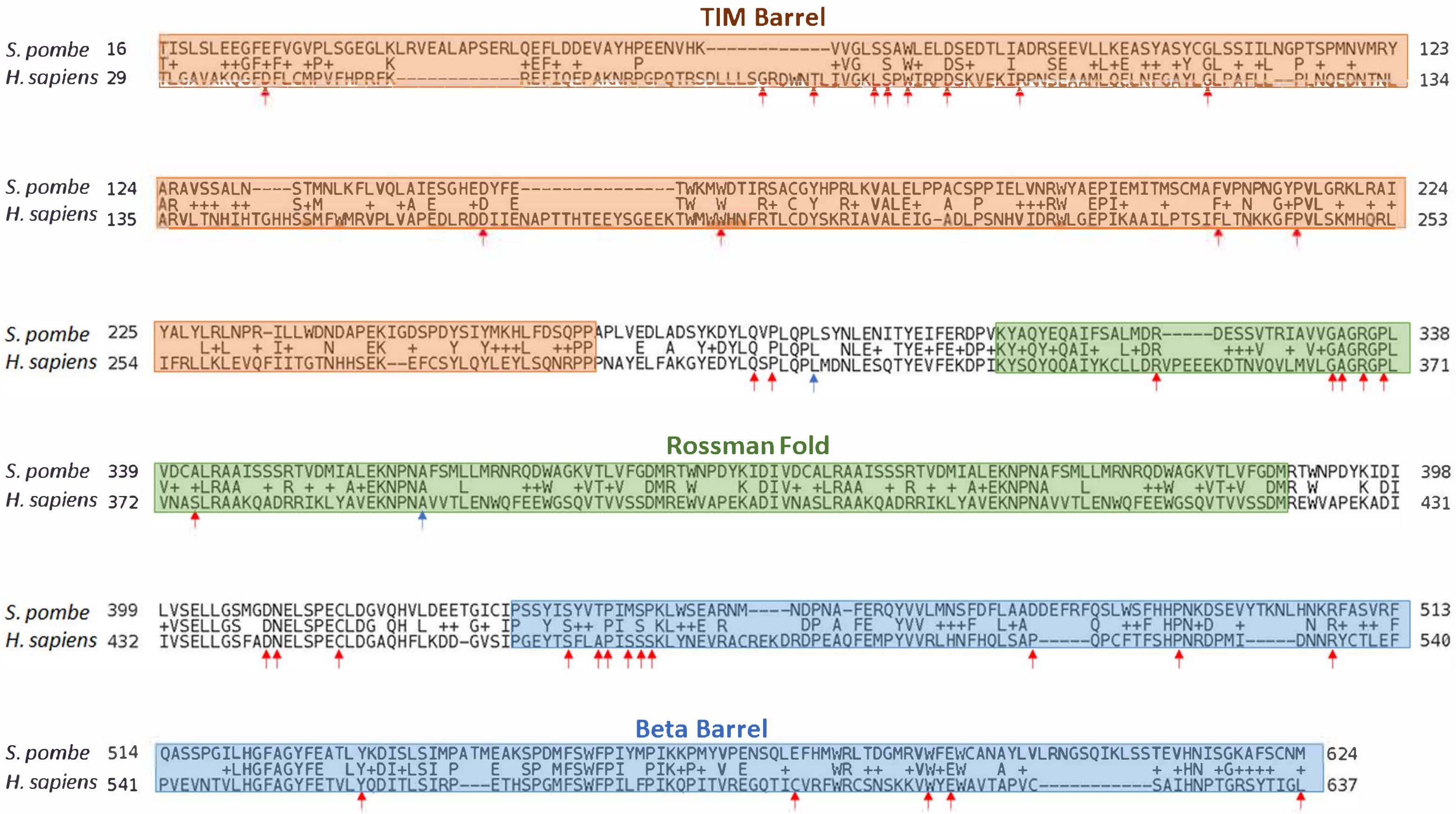

Supplement: Supplementary file 1 [file ijms-24-06042-s001.zip › Supplementary Figure S1.pdf]
