# Supplementary material for: Protein Arginine Methyltransferase 5 (PRMT5) Mutations in Cancer Cells
Source: Int J Mol Sci. 2023 Mar 23;24(7):6042. doi: 10.3390/ijms24076042 (PMC10094674; doi:10.3390/ijms24076042)

Supplementary Figure S2. Mutation distribution for all PRMT genes. Shown is distribution of mutation types deposited on COSMIC (top panel) and distribution of coding mutations in all cancers (lower panel).

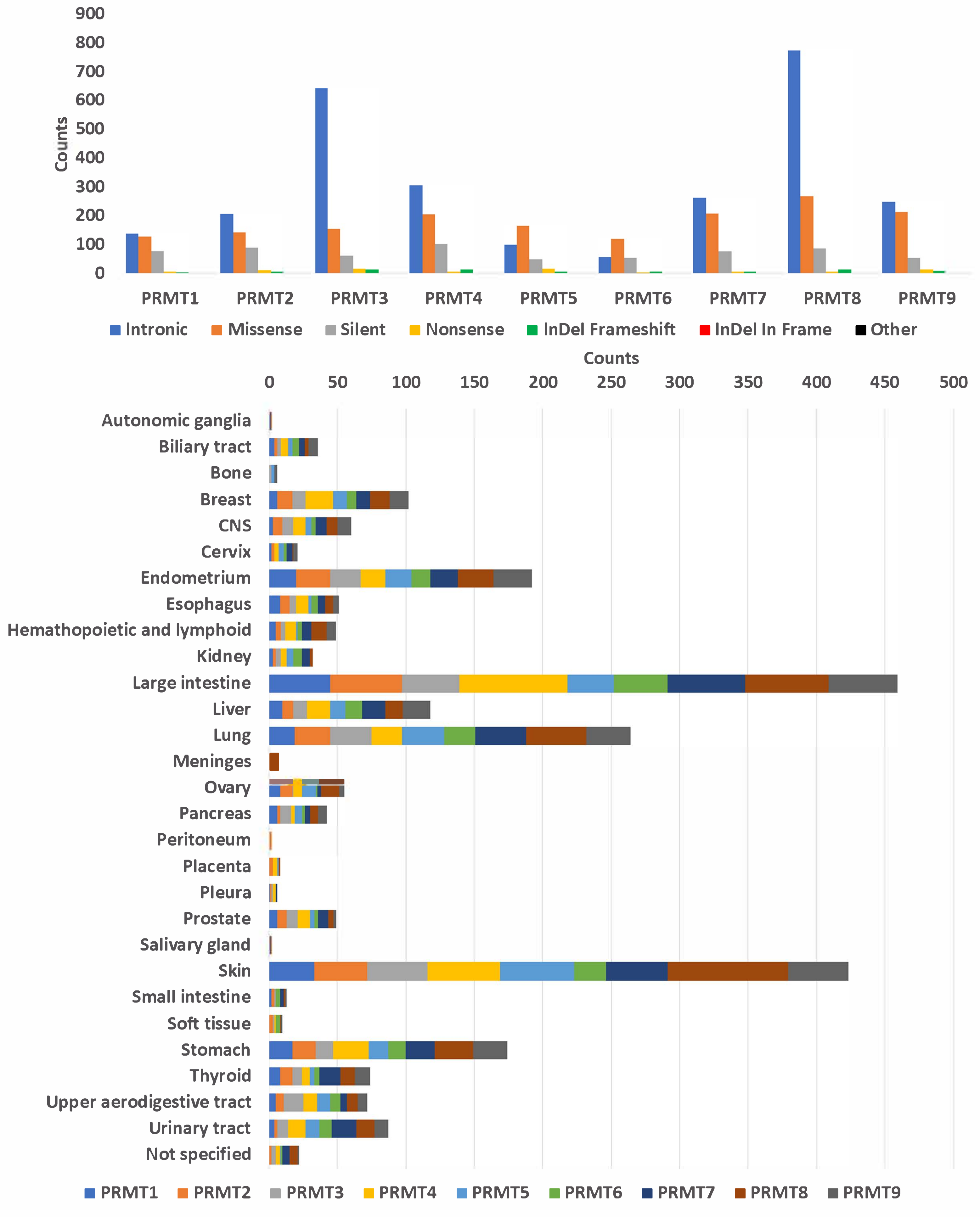

Supplement: Supplementary file 1 [file ijms-24-06042-s001.zip › Supplementary Figure S2.pdf]
