# Supplementary material for: Protein Arginine Methyltransferase 5 (PRMT5) Mutations in Cancer Cells
Source: Int J Mol Sci. 2023 Mar 23;24(7):6042. doi: 10.3390/ijms24076042 (PMC10094674; doi:10.3390/ijms24076042)

Supplementary Figure S4. Amino acid and base pair changes within the coding sequence of PRMT5.

Expanded analysis of data presented in Figure 2. A. 3-D graph of PRMT5 amino acid substitutions. B. Similar graph as in A but showing only those amino acids that have a statistically significant Chi-square deviation from Anoosha et al.

C, D. Distribution of nucleotide changes for all PRMT genes.

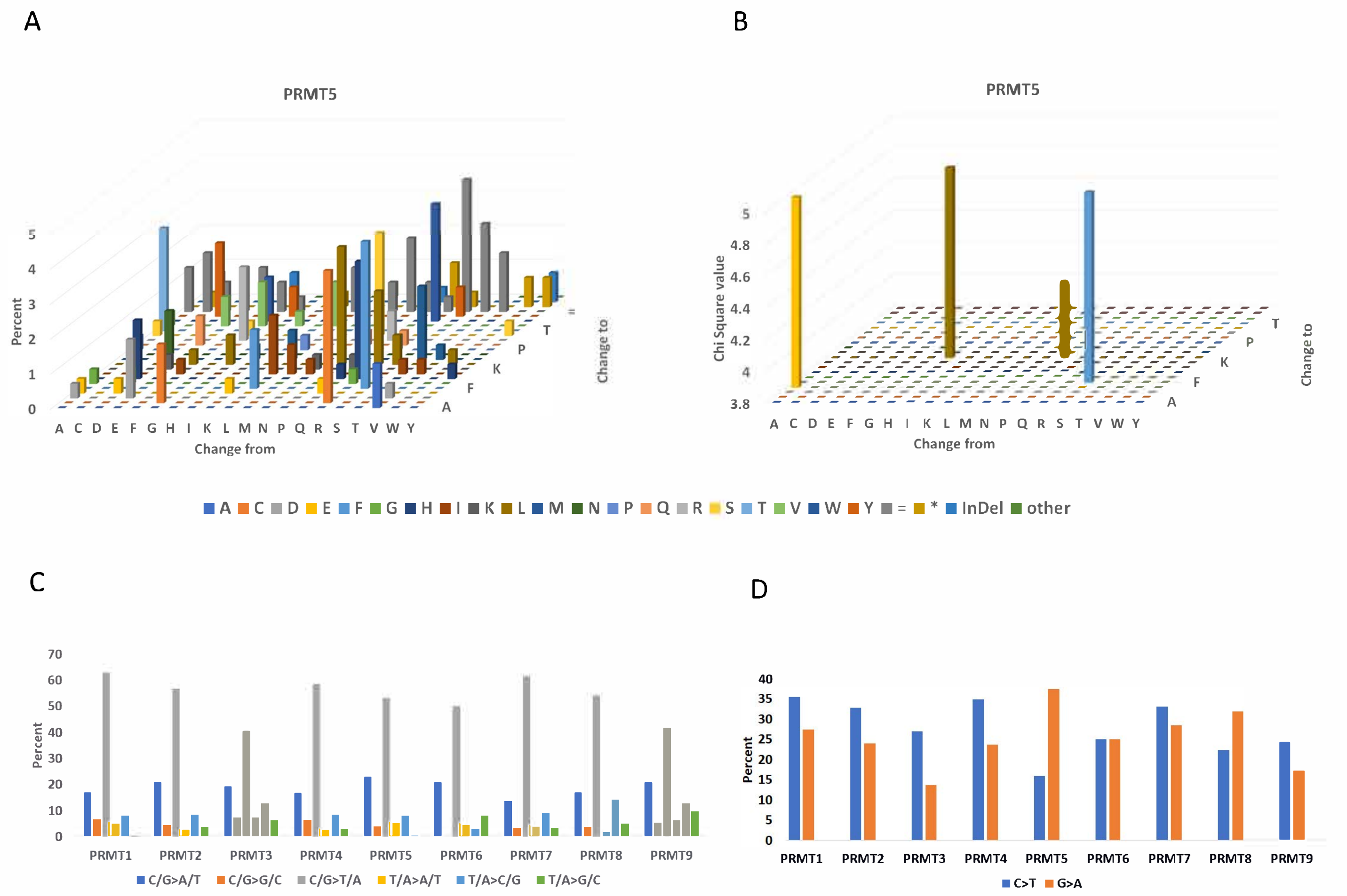

Supplement: Supplementary file 1 [file ijms-24-06042-s001.zip › Supplementary Figure S4.pdf]
