# Supplementary material for: Protein Arginine Methyltransferase 5 (PRMT5) Mutations in Cancer Cells
Source: Int J Mol Sci. 2023 Mar 23;24(7):6042. doi: 10.3390/ijms24076042 (PMC10094674; doi:10.3390/ijms24076042)
Supplement: Supplementary file 1 [file ijms-24-06042-s001.zip › Supplementary Figure S5.pdf]

Supplementary Figure S5. Base pair substitutions in PRMT5 codons. Mutations are highlighted as follows: G>A, red; C>T purple; all others, blue.

```
    Start (0)
5'  atg gcg gcg atg gcg gtc ggg ggt gct ggt ggg agc cgc gtg tcc agc ggg agg gac ctg aat tgc gtc    69
3'  tac cgc cgc tac cgc cag ccc cca cga cca ccc tcg gcg cac agg tcg ccc tcc ctg gac tta acg cag
    Met Ala Ala Met Ala Val Gly Gly Ala Gly Gly Ser Arg Val Ser Ser Gly Arg Asp Leu Asn Cys Val
    1               5                   10                  15                  20

    ccc gaa ata gct gac aca cta ggg gct gtg gcc aag cag ggg ttt gat ttc ctc tgc atg cct gtc ttc    138
    ggg ctt tat cga ctg tgt gat ccc cga cac cgg ttc gtc ccc aaa cta aag gag acg tac gga cag aag
    Pro Glu Ile Ala Asp Thr Leu Gly Ala Val Ala Lys Gln Gly Phe Asp Phe Leu Cys Met Pro Val Phe
        25                  30                  35                  40                  45

    cat ccg cgt ttc aag agg gag ttc att cag gaa cct gct aag aat cgg ccc ggt ccc cag aca cga tca    207
    gta ggc gca aag ttc tcc ctc aag taa gtc ctt gga cga ttc tta gcc ggg cca ggg gtc tgt gct agt
    His Pro Arg Phe Lys Arg Glu Phe Ile Gln Glu Pro Ala Lys Asn Arg Pro Gly Pro Gln Thr Arg Ser
                50                  55                  60                  65

    gac cta ctg ctg tca gga agg gac tgg aat acg cta att gtg gga aag ctt tct cca tgg att cgt cca    276
    ctg gat gac gac agt cct tcc ctg acc tta tgc gat taa cac cct ttc gaa aga ggt acc taa gca ggt
    Asp Leu Leu Leu Ser Gly Arg Asp Trp Asn Thr Leu Ile Val Gly Lys Leu Ser Pro Trp Ile Arg Pro
    70                  75                  80                  85                  90

    gac tca aaa gtg gag aag att cgc agg aac tcc gag gcg gcc atg tta cag gag ctg aat ttt ggt gca    345
    ctg agt ttt cac ctc ttc taa gcg tcc ttg agg ctc cgc cgg tac aat gtc ctc gac tta aaa cca cgt
    Asp Ser Lys Val Glu Lys Ile Arg Arg Asn Ser Glu Ala Ala Met Leu Gln Glu Leu Asn Phe Gly Ala
            95                  100                 105                 110                 115

    tat ttg ggt ctt cca gct ttc ctg ctg ccc ctt aat cag gaa gat aac acc aac ctg gcc aga gtt ttg    414
    ata aac cca gaa ggt cga aag gac gac ggg gaa tta gtc ctt cta ttg tgg ttg gac cgg tct caa aac
    Tyr Leu Gly Leu Pro Ala Phe Leu Leu Pro Leu Asn Gln Glu Asp Asn Thr Asn Leu Ala Arg Val Leu
                    120                 125                 130                 135

    acc aac cac atc cac act ggc cat cac tct tcc atg ttc tgg atg cgg gta ccc ttg gtg gca cca gag    483
    tgg ttg gtg tag gtg tga ccg gta gtg aga agg tac aag acc tac gcc cat ggg aac cac cgt ggt ctc
    Thr Asn His Ile His Thr Gly His His Ser Ser Met Phe Trp Met Arg Val Pro Leu Val Ala Pro Glu
        140                 145                 150                 155                 160

    gac ctg aga gat gat ata att gag aat gca cca act aca cac aca gag gag tac agt ggg gag gag aaa    552
    ctg gac tct cta cta tat taa ctc tta cgt ggt tga tgt gtg tgt ctc ctc atg tca ccc ctc ctc ttt
    Asp Leu Arg Asp Asp Ile Ile Glu Asn Ala Pro Thr Thr His Thr Glu Glu Tyr Ser Gly Glu Glu Lys
                165                 170                 175                 180

    acg tgg atg tgg tgg cac aac ttc cgg act ttg tgt gac tat agt aag agg att gca gtg gct ctt gaa    621
    tgc acc tac acc acc gtg ttg aag gcc tga aac aca ctg ata tca ttc tcc taa cgt cac cga gaa ctt
    Thr Trp Met Trp Trp His Asn Phe Arg Thr Leu Cys Asp Tyr Ser Lys Arg Ile Ala Val Ala Leu Glu
    185                 190                 195                 200                 205

    att ggg gct gac ctc cca tct aat cat gtc att gat cgc tgg ctt ggg gag ccc atc aaa gca gcc att    690
    taa ccc cga ctg gag ggt aga tta gta cag taa cta gcg acc gaa ccc ctc ggg tag ttt cgt cgg taa
    Ile Gly Ala Asp Leu Pro Ser Asn His Val Ile Asp Arg Trp Leu Gly Glu Pro Ile Lys Ala Ala Ile
            210                 215                 220                 225                 230

    ctc ccc act agc att ttc ctg acc aat aag aag gga ttt cct gtt ctt tct aag atg cac cag agg ctc    759
    gag ggg tga tcg taa aag gac tgg tta ttc ttc cct aaa gga caa gaa aga ttc tac gtg gtc tcc gag
    Leu Pro Thr Ser Ile Phe Leu Thr Asn Lys Lys Gly Phe Pro Val Leu Ser Lys Met His Gln Arg Leu
                    235                 240                 245                 250

    atc ttc cgg ctc ctc aag ttg gag gtg cag ttc atc atc aca ggc acc aac cac cac tca gag aag gag    828
    tag aag gcc gag gag ttc aac ctc cac gtc aag tag tag tgt ccg tgg ttg gtg gtg agt ctc ttc ctc
    Ile Phe Arg Leu Leu Lys Leu Glu Val Gln Phe Ile Ile Thr Gly Thr Asn His His Ser Glu Lys Glu
        255                 260                 265                 270                 275

    ttc tgc tcc tac ctc caa tac ctg gaa tac tta agc cag aac cgt cct cca cct aat gcc tat gaa ctc    897
    aag acg agg atg gag gtt atg gac ctt atg aat tcg gtc ttg gca gga ggt gga tta cgg ata ctt gag
    Phe Cys Ser Tyr Leu Gln Tyr Leu Glu Tyr Leu Ser Gln Asn Arg Pro Pro Pro Asn Ala Tyr Glu Leu
                280                 285                 290                 295
```

```
ttt gcc aag ggc tat gaa gac tat ctg cag tcc ccg ctt cag cca ctg atg gac aat ctg gaa tct cag   966
aaa cgg ttc ccg ata ctt ctg ata gac gtc agg ggc gaa gtc ggt gac tac ctg tta gac ctt aga gtc
Phe Ala Lys Gly Tyr Glu Asp Tyr Leu Gln Ser Pro Leu Gln Pro Leu Met Asp Asn Leu Glu Ser Gln
300 305 310 315 320

aca tat gaa gtg ttt gaa aag gac ccc atc aaa tac tct cag tac cag cag gcc atc tat aaa tgt ctg   1035
tgt ata ctt cac aaa ctt ttc ctg ggg tag ttt atg aga gtc atg gtc gtc cgg tag ata ttt aca gac
Thr Tyr Glu Val Phe Glu Lys Asp Pro Ile Lys Tyr Ser Gln Tyr Gln Gln Ala Ile Tyr Lys Cys Leu
325 330 335 340 345

cta gac cga gta cca gaa gag gag aag gat acc aat gtc cag gta ctg atg gtg ctg gga gca gga cgg   1104
gat ctg gct cat ggt ctt ctc ctc ttc cta tgg tta cag gtc cat gac tac cac gac cct cgt cct gcc
Leu Asp Arg Val Pro Glu Glu Glu Lys Asp Thr Asn Val Gln Val Leu Met Val Leu Gly Ala Gly Arg
350 355 360 365

gga ccc ctg gtg aac gct tcc ctg cgg gca gcc aag cag gcc gac cgg cgg ata aag ctg tat gct gtg   1173
cct ggg gac cac ttg cga agg gac gcc cgt cgg ttc gtc cgg ctg gcc gcc tat ttc gac ata cga cac
Gly Pro Leu Val Asn Ala Ser Leu Arg Ala Ala Lys Gln Ala Asp Arg Arg Ile Lys Leu Tyr Ala Val
370 375 380 385 390

gag aaa aac cca aat gcc gtg gtg acg cta gag aac tgg cag ttt gaa gaa tgg gga agc caa gtg acc   1242
ctc ttt ttg ggt tta cgg cac cac tgc gat ctc ttg acc gtc aaa ctt ctt acc cct tcg gtt cac tgg
Glu Lys Asn Pro Asn Ala Val Val Thr Leu Glu Asn Trp Gln Phe Glu Glu Trp Gly Ser Gln Val Thr
395 400 405 410

gta gtc tca tca gac atg agg gaa tgg gtg gct cca gag aaa gca gac atc att gtc agt gag ctt ctg   1311
cat cag agt agt ctg tac tcc ctt acc cac cga ggt ctc ttt cgt ctg tag taa cag tca ctc gaa gac
Val Val Ser Ser Asp Met Arg Glu Trp Val Ala Pro Glu Lys Ala Asp Ile Ile Val Ser Glu Leu Leu
415 420 425 430 435

ggc tca ttt gct gac aat gaa ttg tcg cct gag tgc ctg gat gga gcc cag cac ttc cta aaa gat gat   1380
ccg agt aaa cga ctg tta ctt aac agc gga ctc acg gac cta cct cgg gtc gtg aag gat ttt cta cta
Gly Ser Phe Ala Asp Asn Glu Leu Ser Pro Glu Cys Leu Asp Gly Ala Gln His Phe Leu Lys Asp Asp
440 445 450 455 460

ggt gtg agc atc ccc ggg gag tac act tcc ttt ctg gct ccc atc tct tcc tcc aag ctg tac aat gag   1449
cca cac tcg tag ggg ccc ctc atg tga agg aaa gac cga ggg tag aga agg agg ttc gac atg tta ctc
Gly Val Ser Ile Pro Gly Glu Tyr Thr Ser Phe Leu Ala Pro Ile Ser Ser Ser Lys Leu Tyr Asn Glu
465 470 475 480

gtc cga gcc tgt agg gag aag gac cgt gac cct gag gcc cag ttt gag atg cct tat gtg gta cgg ctg   1518
cag gct cgg aca tcc ctc ttc ctg gca ctg gga ctc cgg gtc aaa ctc tac gga ata cac cat gcc gac
Val Arg Ala Cys Arg Glu Lys Asp Arg Asp Pro Glu Ala Gln Phe Glu Met Pro Tyr Val Val Arg Leu
485 490 495 500 505

cac aac ttc cac cag ctc tct gca ccc cag ccc tgt ttc acc ttc agc cat ccc aac aga gat cct atg   1587
gtg ttg aag gtg gtc gag aga cgt ggg gtc ggg aca aag tgg aag tcg gta ggg ttg tct cta gga tac
His Asn Phe His Gln Leu Ser Ala Pro Gln Pro Cys Phe Thr Phe Ser His Pro Asn Arg Asp Pro Met
510 515 520 525

att gac aac aac cgc tat tgc acc ttg gaa ttt cct gtg gag gtg aac aca gta cta cat ggc ttt gcc   1656
taa ctg ttg ttg gcg ata acg tgg aac ctt aaa gga cac ctc cac ttg tgt cat gat gta ccg aaa cgg
Ile Asp Asn Asn Arg Tyr Cys Thr Leu Glu Phe Pro Val Glu Val Asn Thr Val Leu His Gly Phe Ala
530 535 540 545 550

ggc tac ttt gag act gtg ctt tat cag gac atc act ctg agt atc cgt cca gag act cac tct cct ggg   1725
ccg atg aaa ctc tga cac gaa ata gtc ctg tag tga gac tca tag gca ggt ctc tga gtg aga gga ccc
Gly Tyr Phe Glu Thr Val Leu Tyr Gln Asp Ile Thr Leu Ser Ile Arg Pro Glu Thr His Ser Pro Gly
555 560 565 570 575

atg ttc tca tgg ttt ccc atc ctc ttc cct att aag cag ccc ata acg gta cgt gaa ggc caa acc atc   1794
tac aag agt acc aaa ggg tag gag aag gga taa ttc gtc ggg tat tgc cat gca ctt ccg gtt tgg tag
Met Phe Ser Trp Phe Pro Ile Leu Phe Pro Ile Lys Gln Pro Ile Thr Val Arg Glu Gly Gln Thr Ile
580 585 590 595
```

tgt gtg cgt ttc tgg cga tgc agc aat tcc aag aag gtg tgg tat gag tgg gct gtg aca gca cca gtc 1863
aca cac gca aag acc gct acg tcg tta agg ttc ttc cac acc ata ctc acc cga cac tgt cgt ggt cag
Cys Val Arg Phe Trp Arg Cys Ser Asn Ser Lys Lys Val Trp Tyr Glu Trp Ala Val Thr Ala Pro Val
600 605 610 615 620

tgt tct gct att cat aac ccc aca ggc cgc tca tat acc att ggc ctc tag ccc tgc gtg cca agt gtc 1932
aca aga cga taa gta ttg ggg tgt ccg gcg agt ata tgg taa ccg gag atc ggg acg cac ggt tca cag
Cys Ser Ala Ile His Asn Pro Thr Gly Arg Ser Tyr Thr Ile Gly Leu * Pro Cys Val Pro Ser Val
625 630 635 640

cag agc ctt gga agc agc ttc agg ttc tgc tcc tgt agt aca gaa ggt gca gta cat cta tgg gct gtg 2001
gtc tcg gaa cct tcg tcg aag tcc aag acg agg aca tca tgt ctt cca cgt cat gta gat acc cga cac
Gln Ser Leu Gly Ser Ser Phe Arg Phe Cys Ser Cys Ser Thr Glu Gly Ala Val His Leu Trp Ala Val
645 650 655 660 665

att ccc ctt gcc cat cag aga gga gca ttt caa tct gct ttc ctg cct tac atc aag gtg ggc aag gga 2070
taa ggg gaa cgg gta gtc tct cct cgt aaa gtt aga cga aag gac gga atg tag ttc cac ccg ttc cct
Ile Pro Leu Ala His Gln Arg Gly Ala Phe Gln Ser Ala Phe Leu Pro Tyr Ile Lys Val Gly Lys Gly
670 675 680 685 690

tta taa tta att gca ggg ctc aag cca cca atc tat gaa gac ctc agg cca ggg ggt gag gaa tta gtg 2139
aat att aat taa cgt ccc gag ttc ggt ggt tag ata ctt ctg gag tcc ggt ccc cca ctc ctt aat cac
Leu * Leu Ile Ala Gly Leu Lys Pro Pro Ile Tyr Glu Asp Leu Arg Pro Gly Gly Glu Glu Leu Val
695 700 705 710

ctg gat ttg aag cta cgc act cag cct caa gaa ctc cct gga ata tcc ctg aga aca tgg ggt ttg aac 2208
gac cta aac ttc gat gcg tga gtc gga gtt ctt gag gga cct tat agg gac tct tgt acc cca aac ttg
Leu Asp Leu Lys Leu Arg Thr Gln Pro Gln Glu Leu Pro Gly Ile Ser Leu Arg Thr Trp Gly Leu Asn
715 720 725 730 735

gga ttt tca gcc ttt ttc tgt tct tgt ttt gat ggt ttt gtg taa gag gaa ata caa ata aag tta tag 2277
cct aaa agt cgg aaa aag aca aga aca aaa cta cca aaa cac att ctc ctt tat gtt tat ttc aat atc
Gly Phe Ser Ala Phe Phe Cys Ser Cys Phe Asp Gly Phe Val * Glu Glu Ile Gln Ile Lys Leu *
740 745 750 755

End (2289)

ccc ttt act gca 3′ 2289
ggg aaa tga cgt 5′
Pro Phe Thr Ala
760
