# Supplementary material for: Protein Arginine Methyltransferase 5 (PRMT5) Mutations in Cancer Cells
Source: Int J Mol Sci. 2023 Mar 23;24(7):6042. doi: 10.3390/ijms24076042 (PMC10094674; doi:10.3390/ijms24076042)

Supplementary Figure S6: CASTp calculated binding pocket of wild type PRMT5 and N318K mutation. Comparison of available binding pocket (red) between wild type (left) and the mutant (right). The site
of mutation is indicated by the yellow ring.

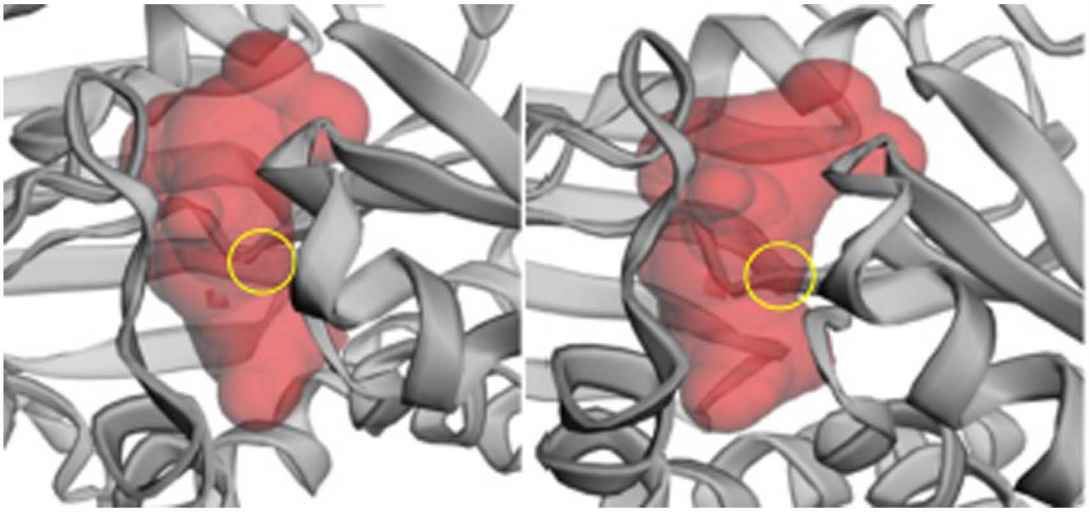

Supplement: Supplementary file 1 [file ijms-24-06042-s001.zip › Supplementary Figure S6.pdf]
