# Supplementary figures and images for: Protein Arginine Methyltransferase 5 (PRMT5) Mutations in Cancer Cells
Source: Int J Mol Sci. 2023 Mar 23;24(7):6042. doi: 10.3390/ijms24076042 (PMC10094674; doi:10.3390/ijms24076042)

Supplementary Figure S3. PRMT5 mutation distribution in cancer tissues.

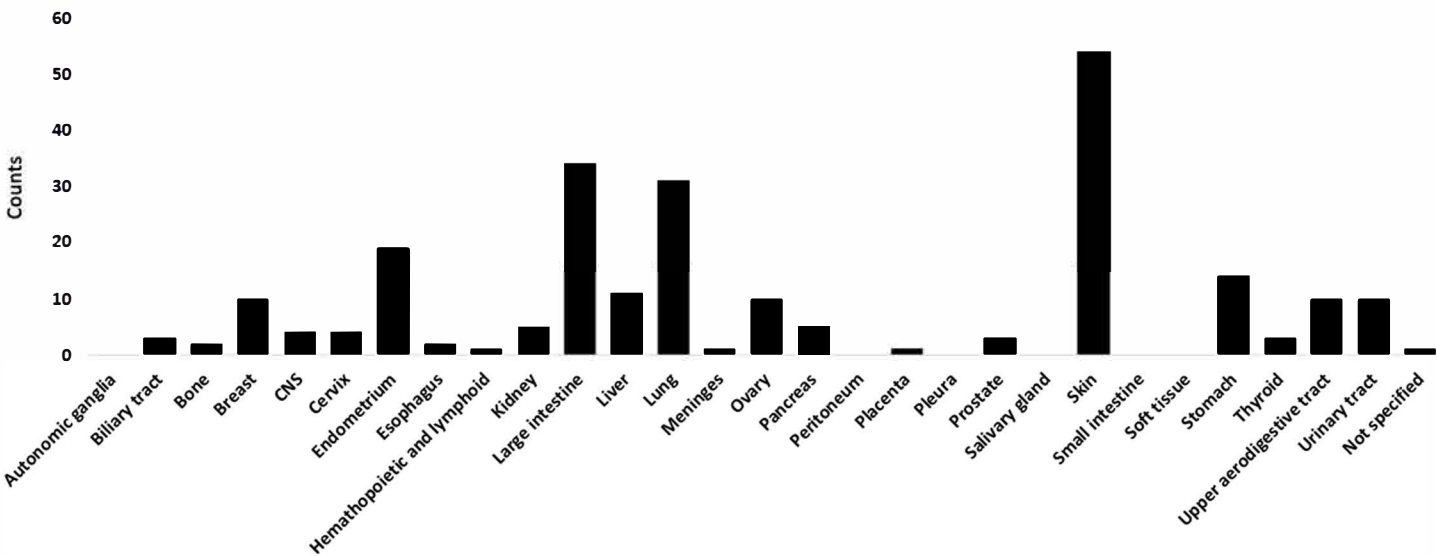

Supplement: Supplementary file 1 [file ijms-24-06042-s001.zip › Supplementary Figure S3.pdf]
